# Neurotoxic effects of dietary glutamate in glaucoma and potential nutritional and pharmacological therapies: a scoping review

**DOI:** 10.1007/s10792-026-04179-4

**Published:** 2026-07-24

**Authors:** Larysse Maria Santiago de Castro, John Hebert da Silva Felix, Larissa Araújo Oliveira Alves, Maria Tayane Barroso dos Santos, Antonio Átila Menezes Ferreira, Thiago Queiroz da Silva, José Cleiton Sousa dos Santos, Aluísio Marques da Fonseca

**Affiliations:** 1 Institute of Engineering and Sustainable Development, University for the International Integration of Afro-Brazilian Lusophony, Redencao, Ceará Brazil; 2 Institute of Exact and Natural Sciences, University for the International Integration of Afro-Brazilian Lusophony, Redencao, Ceará Brazil

**Keywords:** Excitotoxicity, Glaucoma, Glutamate, Glutamine, Neuroprotection

## Abstract

**Purpose:**

To synthesize the available evidence on the relationship between dietary glutamate or glutamatergic metabolism and glaucomatous neurodegeneration, with emphasis on biomarkers, retinal injury mechanisms, and nutritional, antioxidant, or pharmacological strategies with neuroprotective potential.

**Methods:**

This study was conducted as a systematic and bibliometric literature review following the PRISMA 2020 logic of identification, screening, eligibility, and inclusion. Searches were performed in Web of Science, Scopus, and PubMed for studies published in English between 2020 and 2025. The search strategy combined terms related to glaucoma or ocular neurodegeneration, the glutamatergic axis, and biomarkers, mechanisms, or interventions. After screening and full-text assessment, 39 studies were included in the systematic synthesis. Due to methodological heterogeneity, the evidence was synthesized narratively and comparatively, without meta-analysis.

**Results:**

The included studies were organized into six thematic clusters: metabolomic, transcriptomic, and diagnostic biomarkers; pharmacological and neuroprotective interventions; nutritional, antioxidant, and natural-compound neuroprotection; oxidative stress, mitochondrial dysfunction, and regulated cell death; neuroinflammation and glia-mediated retinal injury; and glutamatergic excitotoxicity and neurotransmitter imbalance. The evidence indicates that glutamate-related mechanisms in glaucoma are mainly associated with endogenous glutamatergic metabolism, excitotoxicity, impaired glutamate clearance, glutamate-glutamine homeostasis, oxidative and nitrosative stress, mitochondrial dysfunction, ferroptosis, neuroinflammation, and retinal ganglion cell vulnerability. None of the 39 included studies directly evaluated dietary glutamate or monosodium glutamate as the main exposure.

**Conclusion:**

The available evidence does not support a direct conclusion that dietary glutamate or MSG intake contributes to glaucoma onset or progression. Instead, current findings mainly support an indirect mechanistic relationship between endogenous glutamatergic dysregulation and glaucomatous neurodegeneration. Pharmacological, antioxidant, metabolic, and natural-compound strategies show neuroprotective potential, particularly in experimental models, but clinical and translational studies are still needed to clarify the role of dietary exposure, glutamate-glutamine metabolism, and targeted neuroprotective interventions in glaucoma.

## Introduction

Glutamate is an essential excitatory amino acid widely distributed in nature and plays a crucial role in neurotransmission within the central nervous system (CNS) [1, 2]. Naturally present in a variety of foods, it is also used by the food industry in the form of monosodium glutamate (MSG), primarily as a flavor enhancer [3].

In the CNS, glutamate is the main excitatory neurotransmitter and is essential for processes such as learning and memory. In addition, it is involved in visual function and plays an important role in regulating several brain functions [4, 5]. However, when glutamate levels in the brain become excessive, a phenomenon known as excitotoxicity occurs, which can lead to neuronal death and is associated with various neurodegenerative diseases, including glaucoma [6, 7].

Glaucoma is one of the leading causes of irreversible blindness worldwide, characterized by the progressive degeneration of retinal ganglion cells (RGCs) and the optic nerve [8]. Although elevated intraocular pressure (IOP) is widely recognized as a primary risk factor for glaucoma development, studies suggest that glutamate-mediated excitotoxicity also plays a significant role in the disease pathogenesis [9, 10].

Glutamate toxicity occurs through excessive activation of NMDA (N-methyl-D-aspartate) receptors, leading to calcium influx into retinal cells, which triggers cellular processes such as neuronal death and neuroinflammation [9, 11]. These findings indicate that, in addition to IOP, additional neurodegenerative mechanisms, such as excitotoxicity, should be considered in glaucoma research [12, 13].

Despite advances in understanding the relationship between glutamate and glaucoma, the literature still shows significant gaps regarding effective therapeutic interventions [14, 15]. Previous studies have addressed the impact of glutamate on retinal ganglion cells, but little is known about the effects of dietary glutamate or how nutritional and pharmacological interventions may mitigate glaucoma-associated neurodegeneration [16–18].

Although antioxidant supplementation and NMDA receptor antagonists have shown promising results in experimental models, no recent scoping review has comprehensively synthesized the available evidence on glutamate-related neurodegeneration in glaucoma and the potential role of nutritional and pharmacological interventions [19, 20].

The relevance of this study lies in the growing need for alternative approaches to glaucoma management, particularly in cases of normal-tension glaucoma (NTG), in which conventional therapies based on lowering intraocular pressure (IOP) are not effective [21–24]. In addition, emerging dietary and pharmacological interventions, such as the use of antioxidants, ferroptosis inhibitors, and NMDA receptor antagonists, show strong potential to protect retinal cells and slow disease progression [25]. This scoping review contributes to understanding the impact of glutamate in glaucoma and provides a mapping of current research trends, helping to identify areas requiring further investigation and suggesting new directions for future research and therapeutic interventions [26, 27].

### What this scoping review adds

This scoping review adds to the existing literature by specifically mapping the intersection between dietary glutamate, glaucoma-associated ocular neurodegeneration, and nutritional or pharmacological therapeutic strategies. While previous studies and reviews have addressed glutamate-mediated excitotoxicity, retinal ganglion cell damage, or neuroprotective approaches in glaucoma, the relationship between dietary glutamate exposure and glaucoma-related neurodegeneration remains insufficiently synthesized.

By combining a scoping review approach with complementary bibliometric analysis, this study identifies the main mechanistic pathways discussed in the literature, including excitotoxicity, oxidative stress, neuroinflammation, mitochondrial dysfunction, and ferroptosis. In addition, it highlights emerging intervention strategies, such as antioxidant supplementation, omega-3 fatty acids, flavonoids, NMDA receptor antagonists, ferroptosis inhibitors, and metabolic or neuroprotective approaches. Therefore, this review contributes by organizing current evidence, identifying research trends, and pointing out gaps that should guide future clinical and translational studies.

## Methodology

### Review design

This study was conducted as a systematic and bibliometric literature review, following the logic of study identification, screening, eligibility, and inclusion recommended by PRISMA 2020 [28, 29]. Due to the heterogeneity of the study designs, which included clinical, observational, in vivo experimental studies, in vitro assays, omics analyses, pharmacological studies, and translational investigations, the synthesis was narrative and comparative, without meta-analysis [30].

The review aimed to systematize evidence on the relationship between dietary glutamate or glutamatergic metabolism and neurodegeneration in glaucoma, with attention to associated biomarkers, mechanisms of retinal damage, and nutritional or pharmacological strategies with neuroprotective potential. The guiding question was: *What is the relationship between dietary glutamate or glutamatergic metabolism and neurodegeneration in glaucoma, and which biomarkers and nutritional or pharmacological strategies have been investigated as potential translational tools for diagnosis, monitoring, or neuroprotection?*

### Search strategy

The bibliographic search was structured in the Web of Science, Scopus, and PubMed databases [31], considering the title, abstract, and keywords fields. The defined period was from 2020 to 2025, with restrictions to publications in English. These databases were selected to cover peer-reviewed international journals in the fields of ophthalmology, neuroscience, pharmacology, molecular biology, biomarkers, metabolism, and neuroprotective therapies (Table 1).

**Table 1:** General structure of the search strategy

Element	Content
Databases	Web of science, Scopus, and PubMed
Fields searched	Title, abstract, and keywords
Period	2020 to 2025
Language	English
Block 1	Glaucoma OR ocular hypertension OR normal tension glaucoma OR retinal ganglion cell OR optic neuropathy OR retinal neurodegeneration
Block 2	Glutamate OR dietary glutamate OR monosodium glutamate OR MSG OR excitotoxicity OR NMDA OR AMPA OR glutamate receptor OR glutamate transporter OR EAAT OR GLAST OR EAAC1
Block 3	Biomarker OR metabolomic OR proteomic OR oxidative stress OR neuroinflammation OR mitochondrial dysfunction OR ferroptosis OR microbiome OR diet OR nutrition OR antioxidant OR omega-3 OR flavonoid OR polyphenol OR neuroprotection OR memantine
Operators	OR between terms within the same block; AND between the three blocks

The strategy was organized into three main blocks: glaucoma or ocular neurodegeneration; the glutamatergic axis; and biomarkers, mechanisms, or interventions. Terms related to glaucoma, ocular hypertension, retinal ganglion cells, optic nerve, glutamate, dietary glutamate, monosodium glutamate, excitotoxicity, NMDA and AMPA receptors, glutamate transporters, biomarkers, metabolomics, proteomics, oxidative stress, neuroinflammation, mitochondrial dysfunction, ferroptosis, nutrition, antioxidants, flavonoids, polyphenols, and neuroprotection were incorporated.

### Eligibility criteria

Studies published in English between 2020 and 2025 were included if they presented an explicit relationship with glaucoma, ocular hypertension, optic nerve injury, retinal ganglion cells, or models of retinal neurodegeneration related to glaucoma. The presence of at least one component of the glutamatergic axis was also required, such as glutamate, monosodium glutamate, dietary glutamate, excitotoxicity, NMDA or AMPA receptors, glutamate transporters, glutamate metabolism, or glutamatergic signaling.

Studies were also considered eligible when they provided extractable data on metabolic, inflammatory, oxidative, mitochondrial, proteomic, metabolomic, or ocular biomarkers; mechanisms of retinal neurodegeneration; nutritional, antioxidant, metabolic, or pharmacological strategies; or neuroprotective interventions aimed at reducing glutamatergic damage (Table 2).

**Table 2:** Records retrieved by database in the exported corpus

Database	Exported records
Scopus	313
Web of science	248
PubMed	55
Total	616

Studies with no relationship to glaucoma, ocular hypertension, the optic nerve, or retinal ganglion cells were excluded. Studies addressing glutamate in non-ocular neurological diseases without a connection to the retina or glaucoma were also excluded, as were studies exclusively focused on food or bromatological analysis without relation to ocular neurodegeneration; studies restricted to intraocular pressure reduction without analysis of glutamate, biomarkers, neuroprotection, or neurodegeneration; formulations unrelated to biomarkers or excitotoxicity; narrative reviews, editorials, letters, book chapters, protocols, conference abstracts, and preprints; articles without full text; duplicates; and studies with insufficient methodology for extraction of the required data.

### Study selection

The retrieved records were combined into a single spreadsheet and standardized according to title, authors, year, journal, DOI, abstract, keywords, document type, language, and indexing database. Duplicate removal was performed primarily using the DOI. When the DOI was absent or inconsistent, duplication was verified by comparing title, year, and authorship.

In total, 377 records were assessed. As the initial stage aimed for greater specificity, only studies with explicit adherence to glaucoma or directly related models, to the glutamatergic axis, and to at least one biomarker, mechanism, or intervention dimension were retained. In this initial screening, 45 articles were classified as potentially eligible and 332 were excluded. Subsequently, the available PDFs were assessed in full text, resulting in 42 articles submitted to complete screening.

During full-text reading, each PDF was assessed for adherence to the guiding question, the presence of outcomes related to glaucoma or retinal neurodegeneration, the relationship with glutamate, excitotoxicity, or glutamatergic neurotransmission, the presence of biomarkers or pathophysiological mechanisms, and the availability of extractable data. At the end of the complete screening, 39 articles were included and 3 were excluded.

### Data extraction

Data extraction was performed using a standardized spreadsheet. For each included study, the following information was collected: document ID, authors, title, year, journal, abstract, DOI, thematic cluster, final decision, reason for inclusion, glutamatergic axis, experimental model or population, biomarkers, intervention or exposure, neurodegenerative outcome, key finding, and relationship with the guiding question.

The presence of dietary glutamate or monosodium glutamate was recorded separately. When the study did not directly assess MSG or dietary glutamate, inclusion was justified only when there was a clear connection with glutamatergic metabolism, excitotoxicity, NMDA/AMPA receptors, glutamate/glutamine, glutamate transporters, related biomarkers, or neuroprotective interventions associated with glutamatergic damage (Table 3).

**Table 3:** Summary of the study identification and selection process

Stage	Quantity
Articles pre-included based on title, abstract, and keywords	45
PDFs assessed during complete screening	42
Articles excluded after full-text reading	3
Articles included in the systematic synthesis	39

### Assessment of methodological quality and data reliability

The assessment of methodological quality was qualitative. The following aspects were considered: clarity of the experimental model or population, definition of the type of glaucoma or retinal injury, presence of methods compatible with the study objectives, description of the biomarkers or mechanisms assessed, coherence between results and conclusions, and feasibility of extracting data relevant to the review. No single numerical score was applied, as the studies presented heterogeneous designs, including clinical cohorts, metabolomic or transcriptomic analyses, animal models of ocular hypertension, NMDA-induced injury models, cellular studies, pharmacological interventions, and natural compounds.

### Data synthesis

The synthesis was conducted in a narrative and comparative manner. The included articles were organized according to the main focus of each study, without using this organization as an eligibility criterion. The thematic clusters were defined to structure the article discussion and correspond to the proposed subtitles for the Discussion section.

## Results

### Bibliometric overview

**Fig. 1:**
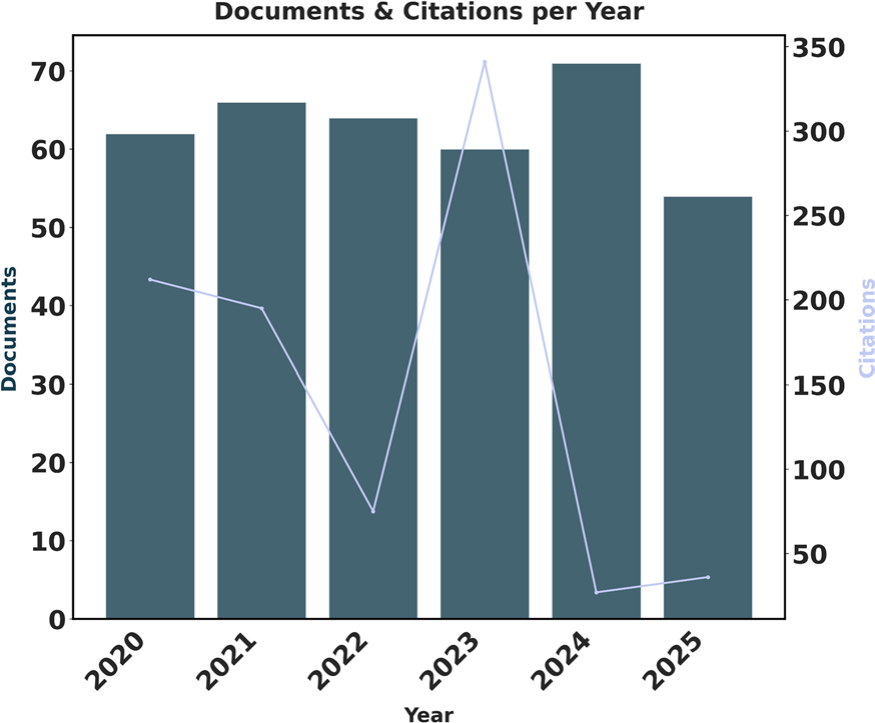
Annual document production and citations by year

The analyzed corpus comprised 377 records published between 2020 and 2025. Scientific production remained relatively stable during the period, with 62 documents in 2020, 66 in 2021, 64 in 2022, 60 in 2023, 71 in 2024, and 54 in 2025. The highest document volume occurred in 2024, whereas 2025 should be interpreted with caution, as it may reflect incomplete indexing for the ongoing year, as shown in Fig. 1.

**Fig. 2:**
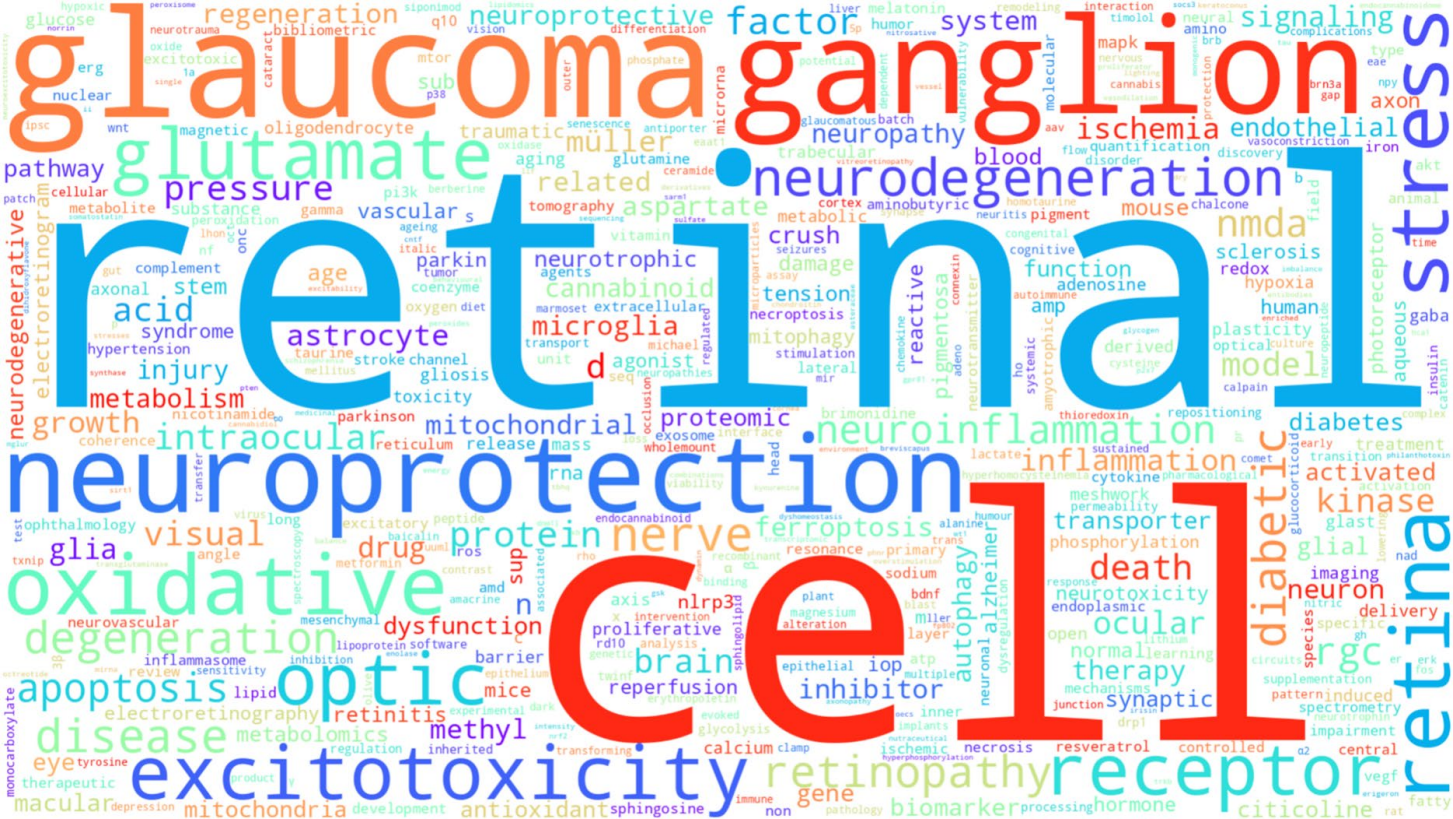
Word cloud generated from recurrent terms in the corpus

In Fig. 2, the word cloud indicated a predominance of terms associated with retina, cell, glaucoma, neuroprotection, oxidative, glutamate, ganglion, stress, optic, receptor, excitotoxicity, degeneration, neuroinflammation, apoptosis, and retinal. This pattern shows that the retrieved literature is concentrated on retinal ganglion cell injury, excitotoxicity mechanisms, and neuroprotective strategies related to glaucoma.

**Fig. 3:**
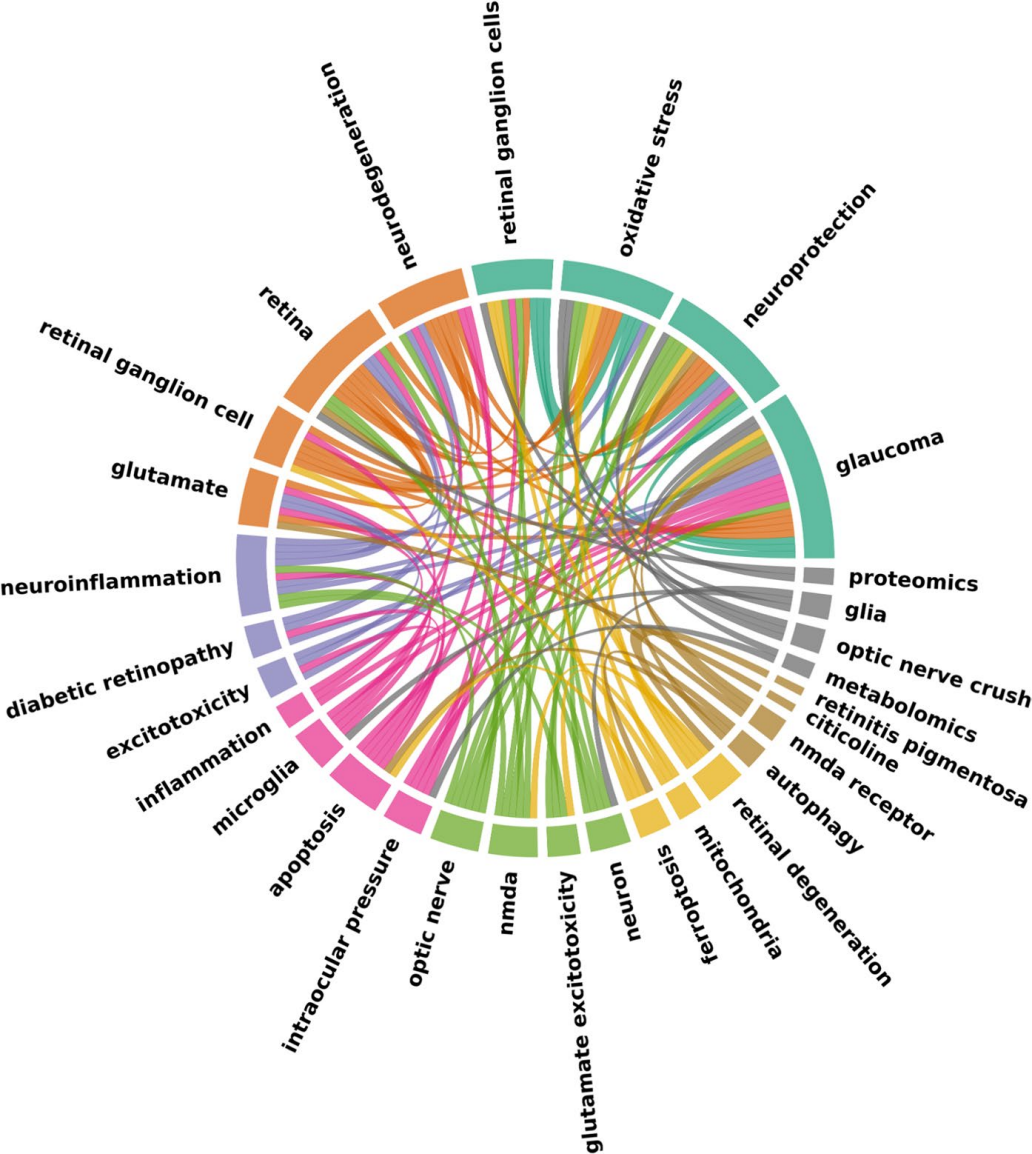
Keyword co-occurrence network of the analyzed corpus

The circular co-occurrence network shown in Fig. 3 reinforced the centrality of glaucoma, neuroprotection, oxidative stress, retinal ganglion cells, glutamate, excitotoxicity, neuroinflammation, and apoptosis. The connection among these terms suggests that the retrieved studies relate the glutamatergic axis to mechanisms of neuronal damage, glial inflammation, regulated cell death, oxidative stress, and neurotransmission alterations.

### Study selection according to the PRISMA flow

Study selection followed the flow of identification, initial screening, full-text reading, and final inclusion. A total of 616 records from Scopus, Web of Science, and PubMed were assessed. During screening by title, abstract, and keywords, 571 records were excluded, including duplicates and records without clear adherence to the predefined criteria, leaving 45 potentially eligible articles.

In the full-text stage, 42 PDFs were assessed. Three studies were excluded because they did not fully meet the review criteria, mainly due to insufficient relationship with glaucoma, the glutamatergic axis, biomarkers, neuroprotection, or mechanisms of retinal neurodegeneration. In the end, 39 articles were included in the systematic synthesis (Table 4).

**Table 4:** Thematic organization of the articles included in the systematic synthesis

Cluster	Synthesis axis	n
1	Metabolomic, Transcriptomic, and Diagnostic Biomarkers	12
2	Neuroprotective and Pharmacological Interventions	7
3	Nutritional, Antioxidant, and Natural-Compound Neuroprotection	8
4	Oxidative Stress, Mitochondrial Dysfunction, and Regulated Cell Death	8
5	Neuroinflammation and Glial-Mediated Retinal Injury	2
6	Glutamatergic Excitotoxicity and Neurotransmitter Imbalance	2
Total		39

### Characterization of the included studies

The 39 included studies were organized into six thematic clusters, defined according to the predominant focus of each article. The largest group consisted of studies on metabolomic, transcriptomic, and diagnostic biomarkers, followed by studies on nutritional neuroprotection or natural compounds, mechanisms of oxidative stress and regulated cell death, and pharmacological or neuroprotective interventions. The smaller clusters included studies directly related to glia-mediated injury and neuroinflammation, as well as glutamatergic alterations and neurotransmitter imbalance.

**Fig. 4:**
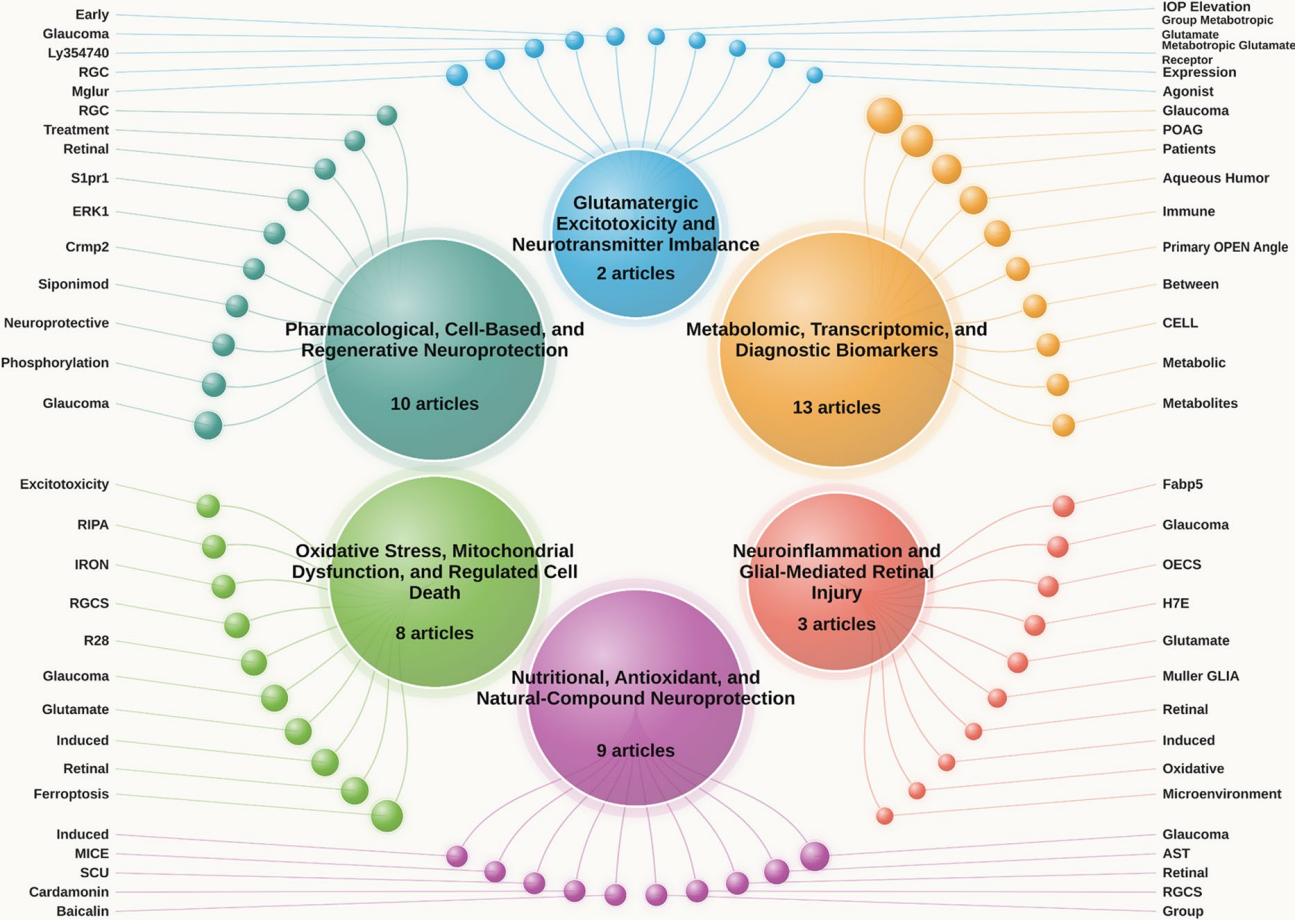
Thematic organization of the articles into clusters and associated terms

Figure 4 presents the thematic organization used to structure the discussion. Since the figure represents the initial visual classification of the articles by theme, the final numbers after full-text reading were consolidated in Table 5. This distinction was maintained to prevent the graphical organization from replacing the final count obtained after complete screening.

**Table 5:** Distribution of included studies by thematic cluster

Cluster	Main focus	n
1	Metabolomic, transcriptomic, and diagnostic biomarkers	12
2	Pharmacological and neuroprotective interventions	7
3	Nutritional, antioxidant, and natural-compound neuroprotection	8
4	Oxidative stress, mitochondrial dysfunction, and regulated cell death	8
5	Neuroinflammation and glia-mediated retinal injury	2
6	Glutamatergic excitotoxicity and neurotransmitter imbalance	2
Total		39

### Thematic synthesis of the clusters

Cluster 1 comprised studies focused on biomarkers and molecular signatures. These articles addressed aqueous humor metabolomics, changes in glutamine, lactate, and histamine, transcriptomic profiles, single-cell RNA analysis, neuroimaging, immune biomarkers, and diagnostic tools associated with glaucoma. The main contribution of this group was to connect metabolic, inflammatory, and molecular alterations to glaucomatous neurodegeneration.

Cluster 2 grouped studies on pharmacological and neuroprotective interventions. These studies investigated drugs, therapeutic repositioning, molecular targets, and neuroprotective strategies with effects on retinal ganglion cells, the optic nerve, ERK1/2 and CRMP2 pathways, inflammation, or visual degeneration. Although not all studies directly assessed glutamate, they were retained when there was a relationship with excitotoxicity, neurodegeneration mechanisms, or RGC protection.

Cluster 3 included studies on natural compounds, antioxidants, and nutritional strategies, such as baicalin, cardamonin, astaxanthin, *Lycium barbarum*, fangchinoline, and scutellarin. These studies support the discussion on interventions with antioxidant, anti-inflammatory, or neuroprotective potential in models of glaucoma, ocular hypertension, or glutamatergic damage.

Cluster 4 concentrated studies on oxidative stress, mitochondrial dysfunction, ferroptosis, necroptosis, apoptosis, and regulated cell death. The presence of models involving GLAST knockout, glutamate, R28 cells, RGCs, and p38 MAPK inhibition reinforces the link between glutamatergic dysregulation, excessive oxidative stress, and the vulnerability of retinal ganglion cells.

Cluster 5 comprised studies on neuroinflammation and glia-mediated retinal injury. The included studies addressed Müller cell activation, astroglia, OECs, S100B, MMP-9, MCP-1, and the glutamatergic microenvironment, supporting the interpretation that glia participate in both the maintenance and disruption of glutamate-glutamine homeostasis in glaucomatous contexts.

Cluster 6 included the studies most directly related to glutamatergic excitotoxicity and neurotransmitter imbalance. These articles addressed early functional alterations in experimental glaucoma, dysregulation of GABAergic and glutamatergic systems, metabotropic glutamate receptors, and modulation of retinal ganglion cell excitability.

### Dietary glutamate

None of the 39 included studies directly evaluated dietary glutamate or monosodium glutamate as the main exposure. Thus, the available evidence was mainly concentrated on glutamatergic metabolism, excitotoxicity, glutamate receptors and transporters, the glutamate-glutamine relationship, mechanisms of retinal damage, and pharmacological, antioxidant, or natural interventions with neuroprotective potential.

This finding indicates an important gap regarding the central theme of the article: although there is relevant evidence on glutamate, excitotoxicity, and glaucomatous neurodegeneration, the specific connection between dietary glutamate or MSG intake and glaucoma progression has not yet been directly explored in the included studies. Therefore, the final synthesis should clearly distinguish direct evidence on dietary glutamate, which was not found, from indirect evidence on glutamatergic metabolism and neurodegenerative damage in glaucoma.

## Discussion

### Neurodegeneration mechanisms associated with glutamate

Glutamate has been extensively investigated as a mediator of ocular neurodegeneration in glaucoma, particularly due to its ability to induce excessive activation of NMDA receptors in retinal ganglion cells (RGCs). This overactivation triggers a cascade of cellular events, ultimately leading to excitotoxic neuronal death [11, 13, 32].

In addition, oxidative stress is identified as a central mechanism, with studies showing that excess glutamate contributes to the generation of reactive oxygen species (ROS), promoting damage to retinal cells [33, 34]. Neuroinflammation, characterized by the activation of microglia and astrocytes, is also an important pathway associated with cellular degeneration in glaucoma [35–37].

Another relevant mechanism involves mitochondrial dysfunction, in which increased glutamate levels disrupt energy production and exacerbate lipid peroxidation, compromising cell viability [38]. In Cluster 4, this mechanism was expanded by studies showing that glutamate/NMDA-related injury is not limited to mitochondrial impairment, but also involves oxidative stress, ferroptosis, necroptosis, apoptosis, and regulated cell death. Experimental models based on glutamate exposure, NMDA-induced retinal injury, GLAST deficiency, and ocular hypertension suggest that impaired glutamate clearance or excitotoxic stimulation may converge on iron accumulation, lipid peroxidation, reduced GPX4/SLC7A11 activity, mitochondrial depolarization, and retinal ganglion cell loss. These findings indicate that downstream targets, such as p38 MAPK, RIP1/RIP3/MLKL, ferroptosis pathways, iron regulation, m6A/Mettl3 signaling, and parkin-dependent mitophagy, may be relevant for neuroprotective strategies in glaucoma [39–46].

In response to these processes, different therapeutic approaches have been proposed, including NMDA receptor antagonists, antioxidant supplementation, and ferroptosis inhibitors. This synthesis highlights the convergence between pathological mechanisms and intervention strategies, reinforcing the potential of neuroprotective therapies as a complement to conventional treatment based on intraocular pressure reduction.

### Therapeutic strategies investigated

Several therapeutic strategies have been investigated to mitigate glutamate-induced damage in retinal ganglion cells. The main approaches include antioxidant supplementation, involving compounds such as omega-3 fatty acids and flavonoids, which have been widely studied due to their neuroprotective effects [47, 48]. These compounds help reduce oxidative stress and support retinal cell health, and studies suggest that antioxidant-rich diets may protect retinal ganglion cells and slow glaucoma progression [49–51].

Another investigated approach involves NMDA receptor antagonists, such as memantine and other compounds, which block excessive glutamate receptor activation, preventing calcium influx into cells and reducing neuronal death [52]. Finally, ferroptosis inhibitors, such as ferrostatin-1, have been shown to protect retinal ganglion cells by reducing glutamate-induced damage. Ferroptosis, which is associated with lipid peroxidation, is a relevant cell-death pathway in the context of glaucoma [53, 54].

In Cluster 2, pharmacological neuroprotection was mainly investigated through strategies that modulate excitotoxic, apoptotic, glial, and pressure-related pathways rather than through direct dietary glutamate exposure. These studies included approaches such as dual NMDA antagonism and intraocular pressure reduction, modulation of S1PR1/Akt/ERK1/2 signaling, reduction of D-serine as an NMDA receptor co-agonist, inhibition of CRMP2 phosphorylation, enhancement of glutamate clearance through GLT-1/EAAT2, and conventional hypotensive agents with additional redox or glutamate-related effects. Overall, this cluster suggests that pharmacological neuroprotection in glaucoma is moving from pressure-centered approaches toward multimodal strategies targeting excitotoxicity, apoptosis, oxidative stress, glial responses, and retinal ganglion cell survival [55–61].

In Cluster 3, nutritional, antioxidant, and natural-compound interventions were mainly explored as modulators of secondary pathways involved in glaucomatous neurodegeneration. Compounds such as astaxanthin, scutellarin, baicalin, cardamonin, fangchinoline, *Lycium barbarum*, and fermented *Pentaclethra macrophylla* showed protective effects in preclinical models involving NMDA/glutamate excitotoxicity, GLAST deficiency, oxidative stress, inflammation, or ocular hypertension. These findings support the potential role of antioxidant and anti-inflammatory strategies as adjunctive neuroprotective approaches; however, they remain predominantly preclinical and do not provide direct evidence that dietary glutamate or MSG intake contributes to glaucoma progression [62–69].

### Dietary glutamate, endogenous glutamatergic metabolism, and retinal excitotoxicity

Glutamate is an amino acid that can be synthesized by the body but is also obtained through the diet. In metabolism, it functions as an essential excitatory neurotransmitter, playing a fundamental role in neurotransmission, protein synthesis, and energy production [70, 71]. However, in the studies included in this review, the available evidence was not centered on dietary glutamate or monosodium glutamate exposure, but rather on endogenous glutamatergic metabolism, excitotoxicity, glutamate transport, glutamate-glutamine cycling, and retinal neurodegeneration.

It is necessary to distinguish dietary glutamate exposure from retinal excitotoxicity mediated by endogenous glutamate. Dietary glutamate, including monosodium glutamate used as a food additive, is extensively metabolized in the gastrointestinal tract, mainly by the intestinal mucosa, reducing its direct systemic availability and making it difficult to establish an immediate causal relationship between dietary intake and glutamate accumulation in retinal tissue [72, 73].

By contrast, retinal excitotoxicity corresponds to a local pathological process characterized by increased endogenous extracellular glutamate or inadequate glutamate clearance in the retinal microenvironment. This condition may promote excessive activation of glutamatergic receptors, intracellular calcium overload, synaptic dysfunction, neuroinflammatory responses, and functional impairment or death of retinal ganglion cells, mechanisms already described in experimental models of glaucoma [74, 75]. Thus, dietary glutamate should be understood as a possible exposure or modulatory factor, whereas glutamate-mediated excitotoxicity represents a local mechanism directly associated with glaucomatous neurodegeneration.

Evidence from Cluster 1 indicates that glaucoma-related biomarkers are not organized around a single isolated marker, but rather around a multicompartimental molecular signature involving aqueous humor, peripheral blood, retina, visual pathways, and cellular models of excitotoxicity. Metabolomic studies showed alterations in glutamine, kynurenine, lactate, histamine, acyl-carnitines, lysophosphatidylcholines, and other metabolic or inflammatory markers, while transcriptomic and neuroimaging studies reinforced the relationship between glutamatergic signaling, calcium pathways, immune remodeling, and retinal or trans-synaptic neurodegeneration. Importantly, some studies reported increased glutamine without a consistent increase in free glutamate, suggesting that glutamine may reflect a buffering or compensatory mechanism within glutamate-glutamine homeostasis [76–86]. This reinforces the relevance of glutamine not as an isolated marker, but as part of the glutamate-glutamine cycle, which may reflect metabolic buffering, glial regulation, and altered excitatory neurotransmission in glaucomatous neurodegeneration.

Cluster 6 further supports the idea that glutamatergic dysfunction may occur early in glaucoma, before or in parallel with marked structural loss of retinal ganglion cells. Experimental studies showed disruption of GABAergic and glutamatergic systems, altered expression of genes related to glutamate-glutamine cycling and glutamate receptors, and functional impairment associated with mGluR II, AMPA-mediated currents, Glul, Gria2, Grm2, and Grm6. These findings indicate that glaucomatous neurodegeneration involves early neurotransmitter imbalance and impaired synaptic regulation, rather than only late-stage neuronal death [74, 75].

The included evidence does not support a direct conclusion that dietary glutamate or MSG intake contributes to glaucoma progression. Instead, the findings mainly support an indirect mechanistic relationship involving endogenous glutamatergic metabolism, excitotoxicity, glutamate receptors and transporters, glutamate-glutamine cycling, neurotransmitter imbalance, and retinal neurodegeneration. Therefore, dietary glutamate should be discussed as a relevant gap in the literature, rather than as a directly demonstrated factor associated with glaucomatous neurodegeneration.

Although elevated glutamate levels in the vitreous humor have not been consistently detected in patients with glaucoma, excitotoxicity is still considered a critical factor in retinal ganglion cell (RGC) degeneration [87]. This occurs due to overactivation of N-methyl-D-aspartate (NMDA) receptors, which promote excessive calcium influx into ganglion cells and trigger apoptotic and necrotic pathways, leading to progressive neuronal loss and visual degeneration [9, 88]. In addition, this mechanism may occur independently of increased intraocular pressure (IOP), making it a relevant factor in the pathogenesis of normal-tension glaucoma (NTG) [89].

Under normal conditions, extracellular glutamate is efficiently cleared from the synaptic cleft by excitatory amino acid transporters, particularly GLAST (glutamate aspartate transporter) and EAAC1 (excitatory amino acid carrier 1). These transporters play a crucial role in reducing glutamate toxicity and preventing damage to retinal ganglion cells (RGCs) [90, 91]. However, dysfunction in these clearance mechanisms can lead to glutamate accumulation and activation of neuroinflammatory pathways, exacerbating neuronal damage [92].

In light of this, recent studies have focused on developing therapeutic strategies to mitigate glutamate excitotoxicity and protect retinal ganglion cells. Among these approaches, NMDA receptor blockers stand out because they prevent excessive glutamate receptor activation, thereby reducing excitotoxicity [93–95]. In addition, NMDA receptor overactivation may induce ferroptosis, a specific form of iron-dependent cell death associated with lipid peroxidation, making it a key therapeutic target. Promising strategies include SB202190, which inhibits the p38 MAPK kinase pathway, leading to reduced iron levels, lipid peroxidation, and oxidative stress [41, 96, 97]; Ferrostatin-1, which has been shown to protect retinal ganglion cells, inhibit ferroptosis, and reduce oxidative stress [46]; and Nec-1 (necrostatin-1) and GSK872, which are necroptosis inhibitors, targeting an inflammatory form of cell death. These compounds exhibit neuroprotective effects by blocking different stages of necroptosis and emerge as potential therapeutic targets for retinal neurodegenerative diseases [45, 98].

### Health implications and intervention strategies

Glutamate is an amino acid widely distributed in foods and in the human body, playing a crucial role in excitatory neurotransmission. However, its accumulation in the central nervous system can lead to a phenomenon known as excitotoxicity, which is a critical factor in neurodegenerative diseases [99, 100]. Glaucoma, one of the leading causes of irreversible blindness worldwide, is associated with retinal ganglion cell (RGC) degeneration and increased intraocular pressure (IOP). It has been identified as a disease that may be exacerbated by excitotoxicity [101].

Excitotoxicity occurs when glutamate receptors, mainly NMDA and AMPA receptors, are excessively activated, leading to excessive calcium influx into cells. This results in oxidative stress and cell death through necrosis or apoptosis. Excitotoxicity is also recognized as one of the main mechanisms of neural loss in several central nervous system disorders [102–104]. Thus, excess glutamate triggers inflammatory mechanisms and mitochondrial dysfunction, increasing retinal ganglion cell (RGC) death. Additional aggravating factors include impaired axonal transport and oxidative stress [13, 34, 101]. Studies have shown that tryptophan and ATP metabolism are altered in patients with glaucoma, with excitotoxicity being one of the main contributing factors to retinal cellular damage. In addition, increased inflammatory cytokines have been observed in the aqueous humor of patients with open-angle glaucoma, likely due to excess glutamate [105–107]. As a potential approach to reduce metabolic stress and excitotoxicity, studies suggest that biomarker-based metabolic therapy informed by proteomic profiles, together with supplementation with metabolites such as α-ketoglutarate, may protect the retina from neurodegeneration [108].

In Cluster 5, neuroinflammation and glia-mediated retinal injury were emphasized as key components of glutamate-related glaucomatous damage. The included studies suggest that glial cells may act both as protectors and amplifiers of retinal injury, depending on their ability to regulate the local glutamatergic microenvironment. In experimental glaucoma, olfactory ensheathing cell transplantation improved visual function and axonal preservation without reducing intraocular pressure, probably by increasing glutamine synthetase and EAAT-mediated glutamate clearance and reducing apoptosis of astrocytes and retinal ganglion cells. In parallel, pharmacological inhibition of HDAC8 reduced abnormal Müller glia activation, MMP-9, MCP-1, ERK/JNK signaling, stress-induced glutamate release, gliosis, and retinal ganglion cell death. These findings reinforce that glial regulation of glutamate-glutamine homeostasis and neuroinflammatory signaling may represent a relevant pressure-independent neuroprotective axis in glaucoma [109, 110].

In recent years, several studies have been conducted to develop intervention and prevention strategies due to the relevance of glutamate in glaucoma progression. In addition, diets rich in vitamins C and E, omega-3 fatty acids, and flavonoids have been recommended [49, 111]. Another approach involves pharmacological strategies, such as glutamate receptor blockers, in which NMDA antagonists have been suggested to reduce retinal degeneration. Moreover, modulation of glial activity through GLP-1R agonists is being explored as a potential neuroprotective strategy [27, 112].

### Gaps, limitations, and future directions

Despite advances in understanding the glutamatergic metabolism, excitotoxicity, and glaucomatous neurodegeneration, important limitations remain that restrict the translation of current findings into clinical practice. In the present synthesis, no included study directly evaluated dietary glutamate or monosodium glutamate as the main exposure, which prevents direct conclusions about the influence of dietary glutamate intake on glaucoma risk or progression. Much of the available evidence is derived from animal models or in vitro studies, whereas clinical studies capable of clarifying the influence of dietary glutamate exposure, individual variations—such as age, genetics, metabolism, comorbidities, and gut microbiota—and excitotoxic mechanisms in specific subtypes, such as normal-tension glaucoma, remain scarce [113–115].

In addition, the selective vulnerability of retinal ganglion cells, together with the involvement of mitochondrial dysfunction, neuroinflammation, altered glutamate-glutamine homeostasis, and mediators such as IL-17A, indicates that glaucomatous progression involves multiple cellular and molecular pathways that are not yet fully defined [116–120]. Therefore, future research should prioritize translational and clinical studies, the identification of early biomarkers in aqueous humor and other biological fluids, and personalized therapeutic strategies aimed at modulating glutamate metabolism, glial responses, and retinal inflammation [76, 121]. This approach may contribute to a broader understanding of the neurodegenerative mechanisms involved in glaucoma and support the development of better-grounded preventive and therapeutic strategies.

### Synthesis of findings and scientific implications

The findings of this scoping review highlight the growing scientific interest in glutamate and its relationship with glaucoma, particularly regarding neurodegeneration mechanisms and the therapeutic strategies investigated. Although some approaches, such as NMDA antagonists and antioxidant supplementation, show promising results, the need for more robust clinical studies is evident [94, 122].

In addition, research on normal-tension glaucoma (NTG) and on nutritional strategies to protect retinal ganglion cells needs to be expanded [123–125]. The gaps identified during this review indicate an urgent need for studies on biomarkers and the personalization of treatments. Pharmacological, antioxidant, metabolic, and nutritional strategies remain promising research directions, but their clinical relevance for glutamate-related glaucoma requires further investigation.

### Research priorities

Future studies should prioritize: (1) clinical investigations directly evaluating whether dietary glutamate or monosodium glutamate intake is associated with glaucoma risk or progression; (2) standardized biomarkers of glutamate-related retinal neurodegeneration, including glutamate-glutamine metabolism, oxidative stress, mitochondrial dysfunction, ferroptosis, and neuroinflammation; (3) longitudinal or randomized studies assessing antioxidant, metabolic, pharmacological, and natural-compound neuroprotective interventions; (4) mechanistic studies clarifying how impaired glutamate clearance, ferroptosis, mitochondrial dysfunction, and glial activation contribute to retinal ganglion cell vulnerability; and (5) translational studies integrating dietary exposure, individual metabolic variability, gut microbiome-related factors, and personalized glaucoma management.

## Conclusion

This review shows that the evidence linking glutamate to glaucomatous neurodegeneration is mainly centered on endogenous glutamatergic metabolism, retinal excitotoxicity, glutamate transport, glutamate-glutamine homeostasis, oxidative stress, mitochondrial dysfunction, neuroinflammation, ferroptosis, and regulated retinal ganglion cell death. Although dietary glutamate and monosodium glutamate were included in the guiding question, none of the 39 studies included in the systematic synthesis directly evaluated dietary glutamate or MSG as the main exposure. Therefore, the available evidence does not support a direct conclusion that dietary glutamate intake contributes to glaucoma onset or progression.

The included studies indicate that glutamatergic dysregulation may contribute to retinal ganglion cell vulnerability through mechanisms involving excessive activation of glutamate receptors, impaired glutamate clearance, calcium overload, oxidative and nitrosative stress, mitochondrial damage, glial dysfunction, and altered neurotransmitter balance. In this context, glutamine and the glutamate-glutamine cycle emerge as relevant components of retinal metabolic homeostasis, suggesting that glutamine may function not as an isolated marker, but as part of a broader compensatory or dysregulated metabolic response in glaucoma.

Pharmacological, antioxidant, metabolic, and natural-compound strategies showed neuroprotective potential, particularly in experimental models involving NMDA/glutamate excitotoxicity, ocular hypertension, GLAST deficiency, ferroptosis, neuroinflammation, or mitochondrial dysfunction. However, these findings remain predominantly preclinical and should be interpreted cautiously. Future studies should prioritize clinical and translational designs capable of directly evaluating dietary glutamate or MSG exposure, standardized biomarkers of glutamatergic damage, glutamate-glutamine metabolism, and the efficacy of neuroprotective interventions in glaucoma. Such approaches may help clarify whether dietary factors act as modulators of retinal vulnerability or whether glutamate-related damage in glaucoma is primarily driven by local endogenous mechanisms.

## Data Availability

The data analyzed in this scoping review are available in the articles included in the review. Bibliometric export files, screening spreadsheets, and analysis outputs generated from Web of Science, Scopus, and PubMed are available from the corresponding author upon reasonable request.
